# Trends in Racial/Ethnic Representation Among US Medical Students

**DOI:** 10.1001/jamanetworkopen.2019.10490

**Published:** 2019-09-04

**Authors:** Elle Lett, H. Moses Murdock, Whitney U. Orji, Jaya Aysola, Ronnie Sebro

**Affiliations:** 1 Perelman School of Medicine at the University of Pennsylvania, Philadelphia; 2 Leonard Davis Institute of Health Economics, University of Pennsylvania, Philadelphia; 3 Department of Biostatistics, Epidemiology, and Informatics, University of Pennsylvania, Philadelphia; 4 Division of General Internal Medicine, University of Pennsylvania, Philadelphia; 5 Office of Inclusion and Diversity, Perelman School of Medicine, University of Pennsylvania, Philadelphia; 6 Penn Medicine Center for Health Equity Advancement, Office of the Chief Medical Officer, University of Pennsylvania, Philadelphia; 7 Department of Genetics, University of Pennsylvania, Philadelphia; 8 Department of Radiology, University of Pennsylvania, Philadelphia; 9 Department of Orthopaedic Surgery, University of Pennsylvania, Philadelphia

## Abstract

**Importance:**

With increasing efforts to create a diverse physician workforce that is reflective of the demographic characteristics of the US population, it remains unclear whether progress has been made since 2009, when the Liaison Committee on Medical Education set forth new diversity accreditation guidelines.

**Objective:**

To examine demographic trends of medical school applicants and matriculants relative to the overall age-adjusted US population.

**Design, Setting, and Participants:**

Repeated cross-sectional study of Association of American Medical Colleges data on self-reported race/ethnicity and sex of medical school applicants and matriculants compared with population distribution of the medical school–aged population (20-34 years). Data from US allopathic medical school applicants and matriculants from 2002 to 2017 were analyzed.

**Main Outcomes and Measures:**

Trends were measured using the representation quotient, the ratio of the proportion of a racial/ethnic group in the medical student body to the general age-matched US population. Linear regression estimates were used to evaluate the trend over time for Asian, black, white, Hispanic, American Indian or Alaska Native (AIAN), and Native Hawaiian or Other Pacific Islander medical school matriculants by sex.

**Results:**

The number of medical school applicants increased 53%, from 33 625 to 51 658, and the number of matriculants increased 29.3%, from 16 488 to 21 326, between 2002 and 2017. During that time, proportions of black, Hispanic, Asian, and Native Hawaiian or Other Pacific Islander male and female individuals aged 20 to 34 years in the United States increased, while proportions of white male and female individuals decreased and proportions of AIAN male and female individuals were stable. From 2002 to 2017, black, Hispanic, and AIAN applicants and matriculants of both sexes were underrepresented, with a significant trend toward decreased representation for black female applicants from 2002 to 2012 (representation quotient slope, −0.011; 95% CI, −0.015 to −0.007; *P* < .001).

**Conclusions and Relevance:**

Black, Hispanic, and AIAN students remain underrepresented among medical school matriculants compared with the US population. This underrepresentation has not changed significantly since the institution of the Liaison Committee of Medical Education diversity accreditation guidelines in 2009. This study’s findings suggest a need for both the development and the evaluation of more robust policies and programs to create a physician workforce that is demographically representative of the US population.

## Key Points

**Question:**

Were the Liaison Committee on Medical Education 2009 diversity accreditation guidelines associated with decreased underrepresentation of minorities in medicine?

**Findings:**

In this cross-sectional study of self-reported race/ethnicity of US medical school matriculants from 2002 to 2017, numbers and proportions of black, Hispanic, and American Indian or Alaska Native medical school matriculants increased, but at a rate slower than their age-matched counterparts in the US population, resulting in increased underrepresentation.

**Meaning:**

This study suggests that while absolute numbers of physicians from minority racial/ethnic groups have increased over time, the physician workforce still does not represent the demographic characteristics of the US population.

## Teaser

This cross-sectional study examines trends in racial/ethnic representation among US medical school applicants and matriculants relative to the overall age-adjusted US population.

## Introduction

Over the last decade, there have been sustained efforts to diversify the physician workforce. In 2009, the Liaison Committee on Medical Education (LCME) instituted formal accreditation guidelines that required medical schools to develop programs or partnerships designed to “make admission to medical education more accessible to potential applicants of diverse backgrounds.”^1^ These efforts have largely centered on individuals designated “underrepresented in medicine” (URM), defined by the Association of American Medical Colleges (AAMC) as those racial/ethnic populations that are “underrepresented in the medical profession relative to their numbers in the general population.”^2^

Motivating these efforts is evidence supporting the benefits of a physician workforce that reflects the shifting demographic characteristics of the US population. Demographic representation has been shown to improve health care access for underserved populations, improve the cultural effectiveness of the physician workforce as a whole, and improve medical research and innovation for all populations.^3,4,5^

In response to the LCME diversity guidelines, several studies examining race/ethnicity at the undergraduate, graduate, and faculty level have reported modest improvements in the proportions of URM racial/ethnic and sex groups within medicine over time.^6,7,8^ These studies, however, fail to account for the shifting demographic characteristics of the US population. They serve to demonstrate that the medical workforce has indeed become more diverse, but do nothing to assess changes in representation for underrepresented groups. Therefore, with this analysis, we provide a systematic approach of assessing the racial/ethnic representation of medical school applicants and matriculants relative to the racial/ethnic distribution of the US population. We apply this approach to assess trends in representation longitudinally as well as cross-sectionally to assess state-level variation in the United States.

## Methods

The protocol for our repeated cross-sectional and cross-sectional analysis of public-use data was approved by the institutional review board at the University of Pennsylvania. The need for informed consent was waived because the data were deidentified and publicly available. We followed the Strengthening the Reporting of Observational Studies in Epidemiology (STROBE) reporting guideline.

### Data Sources

We analyzed data from the AAMC on self-reported race/ethnicity and sex for applicants to US allopathic medical schools from 2002 to 2017, as well as total enrollment by state for 2017 to 2018. These data are publicly available in the AAMC FACTS tables by year. Recent years are available at the AAMC website, and historical data are available by request from the AAMC.^9^ Of note, during the study period, from 2012 to 2017, the AAMC instituted a number of modifications to the nomenclature used to define racial/ethnic groups, the most relevant of which we describe here. Prior to 2012, the AAMC categorized individuals as either Hispanic or non-Hispanic, and only the non-Hispanic group was further classified by race, including “more than one race.” After 2012, the AAMC combined Hispanic ethnicity with other races/ethnicities, with the option “multiple race/ethnicity.” To compare the proportion of American Indian or Alaska Native (AIAN), black, Hispanic, Native Hawaiian or Pacific Islander (NHOPI), Asian, and white applicants and matriculants to the corresponding proportions in the US population, we also obtained national and state-level data from the US Census Bureau.

### Statistical Analysis

Our analysis had 2 objectives. First, we determined trends (from 2002-2017) in racial/ethnic distributions by sex of applicants and matriculants to medical schools relative to the US population of similar age. For our second objective, using 2017 to 2018 academic year data, we examined by state the racial/ethnic distributions of matriculants to medical school relative to the state’s racial/ethnic population of similar age.

We used a representation quotient (RQ), defined as the ratio of proportion of a particular subgroup among the total population of applicants or matriculants relative to the corresponding estimated proportion of that subgroup in the US population. An RQ greater than 1 indicates that a subgroup is overrepresented among medical school applicants, matriculants, or enrollees relative to the US population, and an RQ less than 1 indicates a subgroup is underrepresented. The magnitude of the RQ is also interpretable. An RQ of 0.50 indicates that the representation for that subgroup in the medical school applicant, matriculant, or enrollee pool is 50% of that subgroup’s representation in the total US population, and an RQ of 1.75 indicates that the representation of that subgroup is 75% greater than its representation in the US population. We compared the RQ to counts and proportions of each race/ethnicity for medical school applicants and matriculants (presented in eFigures 1-4 in the Supplement) and examined national longitudinal trends for applicants and matriculants from 2002 to 2017. We present longitudinal trends with estimates for linear regression of year on RQ with corresponding *P* values and 95% confidence intervals. All statistical tests were 2-sided and *P* < 3.57 × 10^−3^ was considered statistically significant. We excluded the multiple race/ethnicity category in our analysis, given the changes in categorization described, after conducting a sensitivity analysis with a composite URM category that included the multiple race/ethnicity group to determine how this categorization affected our primary analysis. We also analyzed the 2002 to 2012 and 2013 to 2017 intervals separately, given the different treatment of individuals who self-identify in multiple racial/ethnic subgroups. Using Bonferroni correction, we adjusted for multiple hypothesis testing for each of the 14 possible groups, with a type I error rate per test of 3.57 × 10^−3^, yielding an overall type I error of 0.05 within each interval for both matriculants and applicants.

We then determined cross-sectional variation in state-level RQ estimates for 2017 to 2018 medical school enrollees. We generated cross-sectional analysis choropleth maps of the state-level RQs using the gmap package in R statistical software version 3.6.0 (R Project for Statistical Computing).^10^

We performed our trend analyses in Excel (Microsoft Corp) and R version 3.5.3 and used R version 3.6.0 for the cross-sectional analysis.

## Results

Between 2002 and 2017, the number of medical school applicants increased 53.6%, from 33 625 to 51 658, and the number of matriculants increased 29.3%, from 16 488 to 21 326. In that same interval, the proportion of male and female black, Hispanic, Asian, and NHOPI individuals aged 20 to 34 years in the United States increased. The white male and female proportions decreased, whereas the AIAN male and female proportions remained relatively unchanged.

### Applicants

**Figure 1.**
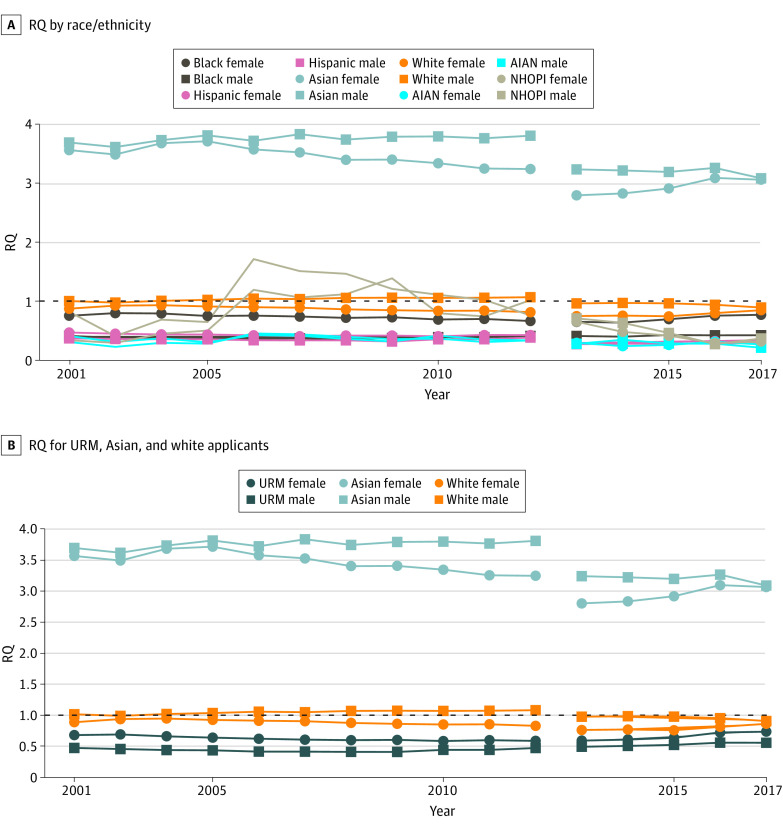
Representation Quotient (RQ) by Race/Ethnicity and Sex for Medical School Applicants, 2002 to 2017 An RQ greater than 1 indicates that a subgroup is overrepresented among medical school applicants relative to the US population, and an RQ less than 1 indicates that a subgroup is underrepresented. AIAN indicates American Indian and Native American; NHOPI, Native Hawaiian or Pacific Islander; and URM, underrepresented in medicine.

In the 2002 to 2012 interval, the number of black and Hispanic applicants of both sexes increased (eFigure 1 in the Supplement); however, the RQ for black female applicants declined from 0.75 to 0.66, and was relatively constant at approximately 0.40 for black male applicants (Figure 1). The slopes and corresponding *P* values (Table) show that the declines in RQ for black female applicants were statistically significant (RQ slope, −0.011; 95% CI, −0.015 to −0.007; *P* < .001), whereas there was no statistically significant increase or decrease in representation among black male applicants (RQ slope, 4.43 × 10^−4^; 95% CI, 3.30 × 10^−3^ to 4.19 × 10^−3^; *P* = .80). During the same interval, the RQ for Hispanic female applicants varied between 0.40 and 0.47 and for Hispanic male applicants between 0.31 and 0.38 (Figure 1), with no statistically significant increase or decrease in RQ for either group (Table). There were no significant trends for NHOPI or AIAN applicants. The mean RQ for male and female AIAN applicants was approximately 0.35 (Figure 1). For NHOPI, the results were more variable, with the RQ for male applicants ranging from 0.29 to 1.39, and for female applicants from 0.4 to 1.7 (Figure 1).

**Table.** Slope of RQ Over Time, 2002 to 2012 and 2013 to 2017

Race/Ethnicity	2002-2012		2013-2017	
	RQ Estimate (95% CI)	*P* Value	RQ Estimate (95% CI)	*P* Value
Applicants				
Black female	−0.011 (−0.015 to −0.007)	<.001^a^	0.036 (0.013 to 0.059)	.02
Black male	4.43 × 10^−4^ (3.30 × 10^−3^ to 4.19 × 10^−3^)	.80	4.85 × 10^−3^ (−5.23 × 10^−3^ to 1.49 × 10^−2^)	.22
Hispanic female	−4.0 × 10^−3^ (−7.05 × 10^−3^ to −9.66 × 10^−4^)	.02	1.178 × 10^−2^ (9.4 × 10^−3^ to 1.41 × 10^−2^)	<.001^a^
Hispanic male	2.74 × 10^−4^ (−4.41 × 10^−3^ to 3.86 × 10^−3^)	.88	4.59 × 10^−3^ (5.6 × 10^−5^ to 9.13 × 10^−3^)	.05
Asian female	−3.97 × 10^−2^ (−5.99 × 10^−2^ to −1.96 × 10^−2^)	<.001^a^	0.07926 (2.52 × 10^−2^ to 1.33 × 10^−1^)	.02
Asian male	1.2 × 10^−2^ (9.2 × 10^−4^ to 2.33 × 10^−2^)	.04	2.59 × 10^−2^ (−8.94 × 10^−2^ to 3.76 × 10^−2^)	.29
White female	−9.86 × 10^−3^ (−1.46 × 10^−2^ to 5.09 × 10^−3^)	.001^a^	2.4 × 10^−2^ (−1.52 × 10^−3^ to 4.95 × 10^−2^)	.06
White male	8.13 × 10^−3^ (5.41 × 10^−3^ to 1.09 × 10^−2^)	<.001^a^	−1.71 × 10^−2^ (−3.67 × 10^−2^ to 2.56 × 10^−3^)	.07
AIAN female	1.2 × 10 (9.2 × 10^−4^ to 2.3 × 10^−2^)	.04	1.79 × 10^−3^ (−3.18 × 10^−2^ to 3.54 × 10^−2^)	.88
AIAN male	−9.90 × 10^−4^ (−1.1 × 10^−2^ to 9.32 × 10^−3^)	.83	−1.98 × 10^−2^ (−6.1 × 10^−2^ to 2.18 × 10)	.23
NHOPI female	3.9 × 10^−2^ (−4.98 × 10^−2^ to 0.129)	.35	8.68 × 10^−2^ (−0.146 to −0.027)	.02
NHOPI male	7.21 × 10^−2^ (6.63 × 10^−3^ to 0.138)	.03	−0.104 (−0.192 to −0.016)	.03
URM female	−1.06 × 10^−2^ (−1.37 × 10^−2^ to −7.43 × 10^−3^)	<.001^a^	4.04 × 10^−2^ (1.91 × 10^−2^ to 6.17 × 10^−2^)	.009
URM male	−9.874 × 10^−4^ (−6.35 × 10^−3^ to 4.37 × 10^−3^)	.69	1.82 × 10^−2^ (8.91 × 10^−3^ to 2.75 × 10^−2^)	.008
Matriculants				
Black female	−9.45 × 10^−3^ (−1.41 × 10^−2^ to −4.84 × 10^−3^)	.001^a^	0.0314 (2.18 × 10^−3^ to 6.06 × 10^−2^)	.04
Black male	3.63 × 10^−4^ (−2.18 × 10^−3^ to 2.91 × 10^−3^)	.76	5.53 × 10^−3^ (−4.84 × 10^−3^ to 1.59 × 10^−2^)	.19
Hispanic female	2.445 × 10^−3^ (8.44 × 10^−4^ to 5.73 × 10^−3^)	.13	5.35 × 10^−2^ (−2.43 × 10^−3^ to 1.31 × 10^−2^)	.12
Hispanic male	5.42 × 10^−3^ (8.57 × 10^−4^ to 9.98 × 10^−3^)	.03	−1.20 (−6.39 × 10^−3^ to 3.99 × 10^−3^)	.52
Asian female	−4.48 × 10^−2^ (−5.92 × 10^−2^ to −3.0 × 10^−2^)	<.001^a^	0.09849 (1.72 × 10^−2^ to 1.80 × 10^−1^)	.03
Asian male	−3.51 × 10^−3^ (−0.0218 to 0.148)	.67	9.49 × 10^−3^ (−0.106 to 0.087)	.77
White female	−7.65 × 10^−3^ (−1.18 × 10^−2^ to −3.46 × 10^−3^)	.003^a^	1.54 × 10^−2^ (−1.0 × 10^−3^ to 3.18 × 10^−2^)	.06
White male	5.884 × 10^−3^ (3.44 × 10^−3^ to 8.33 × 10^−3^)	<.001^a^	−1.82 × 10^−2^ (−3.38 × 10^−2^ to −2.61 × 10^−3^)	.03
AIAN female	1.13 × 10^−2^ (−7.00 × 10^3^ to 2.95 × 10^−2^)	.20	1.26 × 10^−2^ (−3.99 × 10^−2^ to 6.51 × 10^−2^)	.50
AIAN male	−5.36 × 10^−3^ (−2.1 × 10^−2^ to 9.95 × 10^−3^)	.45	−2.32 × 10^−2^ (−6.71 × 10^−2^ to 2.1 × 10^−2^)	.19
NHOPI female	0.020 (−0.040 to 0.081)	.47	−0.108 (−0.206 to −0.010)	.04
NHOPI male	0.044 (−4.2 × 10^−3^ to 0.092)	.07	−0.102 (−0.177 to −0.026)	.02
URM female	−4.99 × 10^−3^ (−8.03 × 10^−3^ to −1.94 × 10^−3^)	.005	3.47 × 10^−2^ (1.23 × 10^−2^ to 5.71 × 10^−2^)	.02
URM male	2.99 × 10^−3^ (−6.1 × 10^−4^ to 6.58 × 10^−3^)	.09	1.43 × 10^−2^ (4.68 × 10^−3^ to 2.39 × 10^−2^)	.02

Abbreviations: AIAN, American Indian/Alaska Native; NHOPI, Native Hawaiian/Other Pacific Islander; RQ, representation quotient; URM, underrepresented in medicine.

^a^ Statistically significant at *P* < 3.57 × 10^−3^.

The proportion of white male and female applicants declined from 2002 to 2012 (eFigure 2 in the Supplement), while the RQ for white male applicants increased from 1.00 to 1.07, and for white female applicants decreased from 0.87 to 0.81. Based on the slopes, the trends toward increased overrepresentation for white male individuals (RQ slope, 8.13 × 10^−3^; 95% CI, 5.41 × 10^−3^ to 1.09 × 10^−2^; *P* < .001) and decreased representation for white female individuals (RQ slope, −9.86 × 10^−3^; 95% CI, −1.46 × 10^−2^ to 5.09 × 10^−3^; *P* = .001) among applicants were statistically significant in the 2002 to 2012 period. The proportion of Asian male and female applicants generally increased from 2002 to 2012 (eFigure 2 in the Supplement), while the representation quotient for both sexes decreased but remained greater than 3, consistent with overrepresentation. In the sensitivity analysis, the RQ for URM male applicants from 2002 to 2012 was between 0.39 and 0.45. The RQ for URM female applicants declined from 0.66 to 0.58 in 2009 and was unchanged for the rest of the period (Figure 1).

In 2013 to 2017, only Hispanic female applicants showed a statistically significant trend in RQ toward increased representation (RQ slope, 1.178 × 10^−2^; 95% CI, 9.4 × 10^−3^ to 1.41 × 10^−2^; *P* < .001) (Table), despite increases in the counts of black and Hispanic applicants of both sexes (eFigure 1 in the Supplement). The RQ for Hispanic female applicants increased from 0.29 to 0.34. For Hispanic male applicants, RQ was relatively constant at approximately 0.28. The RQ for black female applicants increased from 0.65 to 0.77 but was stagnant for black male applicants at approximately 0.42 (Figure 1). The RQ values for AIAN male and female applicants were similar to those in the previous period, ranging from 0.21 to 0.35. The RQs for NHOPI applicants varied, ranging from 0.27 to 0.71 in 2013 to 2017. Asian male and female applicants were overrepresented among applicants in 2013 to 2017, with RQs approximately 3 or greater throughout the period. In the sensitivity analysis for the composite URM category, the RQ for female applicants increased from 0.57 to 0.72. For URM male applicants, RQ increased from 0.47 to 0.54.

### Matriculants

**Figure 2.**
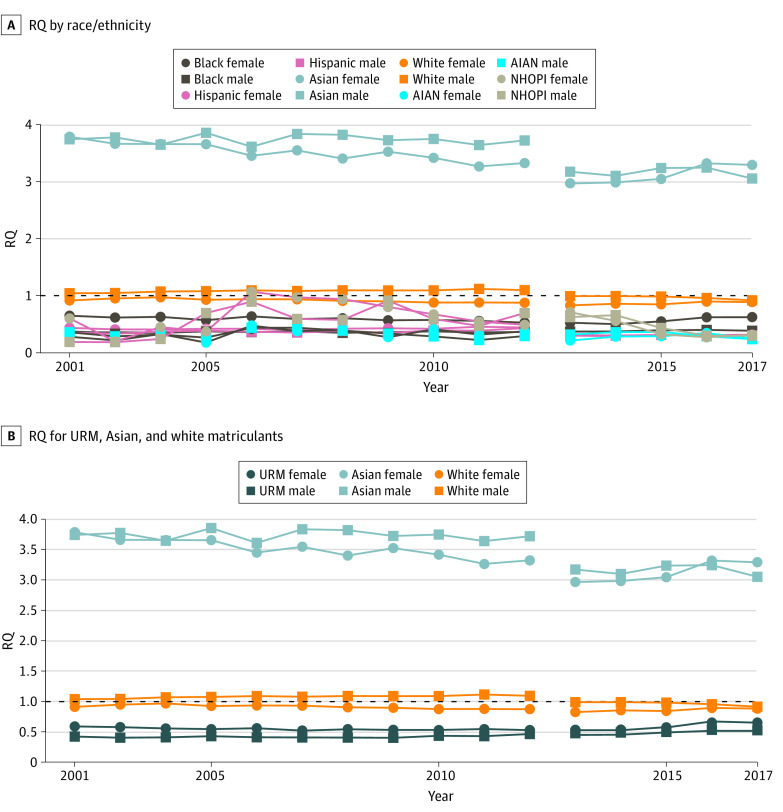
Representation Quotient (RQ) by Race/Ethnicity and Sex for Medical School Matriculants, 2002 to 2017 An RQ greater than 1 indicates that a subgroup is overrepresented among medical school matriculants relative to the US population, and an RQ less than 1 indicates that a subgroup is underrepresented. AIAN indicates American Indian and Native American; NHOPI, Native Hawaiian or Pacific Islander; and URM, underrepresented in medicine.

Counts and proportions of matriculant racial/ethnic subgroups over time are plotted in eFigure 3 and eFigure 4 in the Supplement. From 2002 to 2012, the RQ for black female matriculants declined from 0.65 to 0.53, and the RQ for black male matriculants was unchanged at approximately 0.36 (Figure 2). The slopes and corresponding *P* values (Table) show that the declines in RQ for black female matriculants were statistically significant (RQ slope, −9.45 × 10^−3^; 95% CI, −1.41 × 10^−2^ to −4.84 × 10^−3^; *P* = .001), whereas there was no statistically significant increase or decrease in RQ for black male matriculants during that time (RQ slope, 3.63 × 10^−4^; 95% CI, −2.18 × 10^−3^ to 2.91 × 10^−3^; *P* = .76). During the same interval, the RQs for Hispanic female and male matriculants were similar to applicant numbers, with RQ for Hispanic female matriculants varying between 0.40 and 0.45 and for Hispanic male matriculants between 0.35 and 0.43 (Figure 2). Neither group displayed significant trends (Table). There were no significant increases or decreases for NHOPI and AIAN matriculants. The mean (SD) RQ for male and female AIAN matriculants was 0.32 (0.07) (Figure 2). For NHOPI, RQ for male matriculants ranged from 0.18 and 0.91 and for female matriculants from 0.20 to 1.07 (Figure 2).

The RQ for white male matriculants increased from 1.04 to 1.10, and the RQ decreased for white female matriculants from 0.91 to 0.88 (Figure 2). Trends toward increased overrepresentation for white male matriculants (RQ slope, 5.884 × 10^−3^; 95% CI, 3.44 × 10^−3^ to 8.33 × 10^−3^; *P* < .001) and decreased representation for white female matriculants (RQ slope, −7.65 × 10^−3^; 95% CI, −1.18 × 10^−2^ to −3.46 × 10^−3^; *P* = .003) were statistically significant in the 2002 to 2012 period (Table). The RQ for Asian matriculants of both sexes decreased but remained greater than 3, consistent with overrepresentation, with a significant trend toward decreased representation for Asian female matriculants. In the sensitivity analysis, the RQ remained stagnant at approximately 0.55 for URM female matriculants and 0.42 for URM male matriculants (Figure 2).

From 2012 to 2017, no groups displayed significant increases or decreases in RQ. For Hispanic matriculants of both sexes, RQ was relatively constant at approximately 0.30 (Figure 2). The RQ for black female matriculants increased from 0.52 to 0.62, and for black male matriculants was stagnant at approximately 0.38 (Figure 2). The RQs for AIAN male and female matriculants were similar to those in the previous period, ranging from 0.21 to 0.36. The RQs for NHOPI male and female matriculants varied, ranging from 0.27 to 0.71 in the 2013 to 2017 period. Asian matriculants of both sexes were overrepresented in 2013 to 2017, with RQs of approximately 3 or greater throughout the period. In the sensitivity analysis for the composite URM category, the RQ for female matriculants ranged from 0.54 to 0.66 and the RQ for male matriculants from 0.48 to 0.54.

### State-Level Analysis

**Figure 3.**
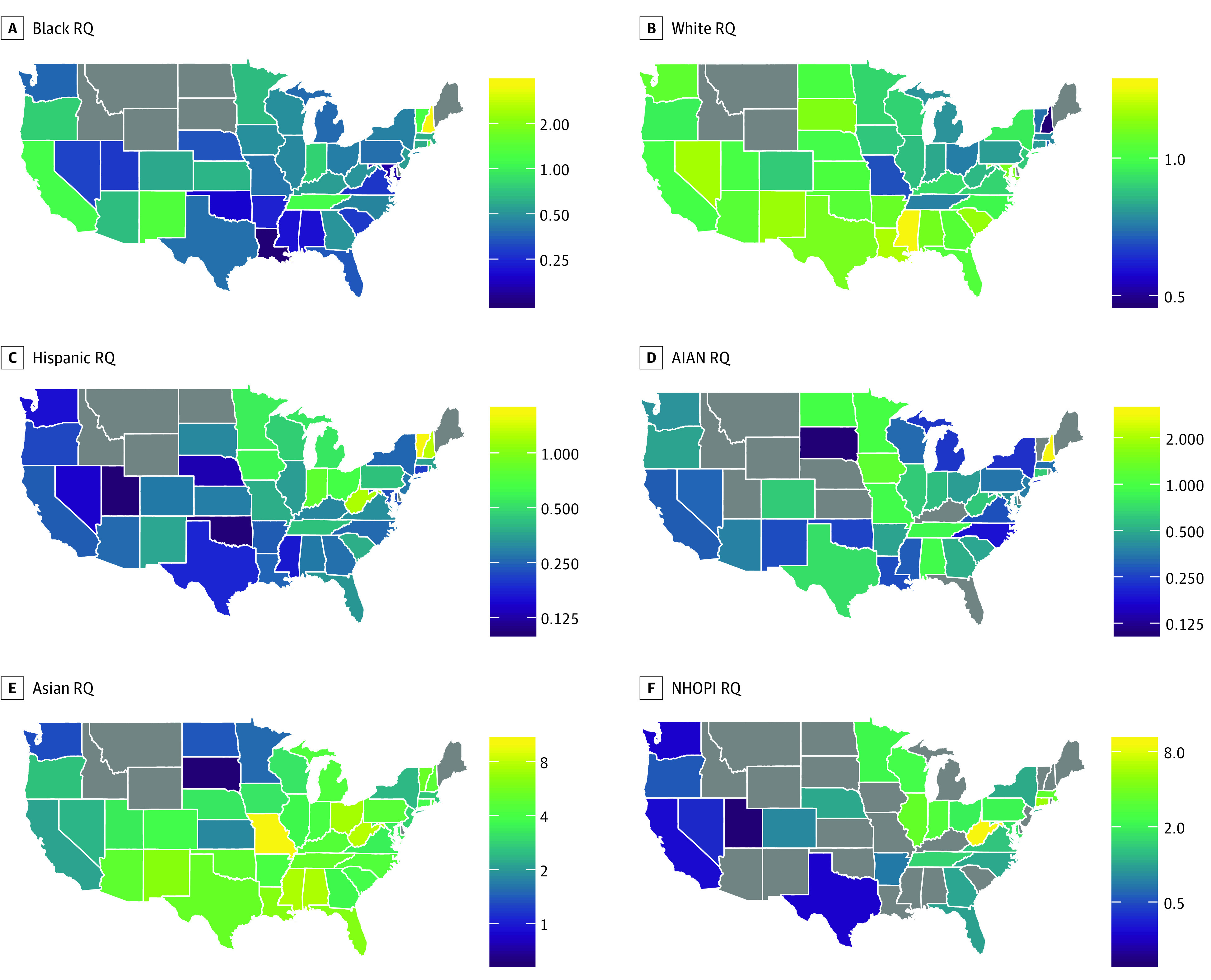
Choropleth Maps of Representation Quotient (RQ) for Medical School Enrollees by Race/Ethnicity and State, 2017 to 2018 An RQ greater than 1 indicates that a subgroup is overrepresented among medical school enrollees relative to the US population, and an RQ less than 1 indicates that a subgroup is underrepresented. AIAN indicates American Indian and Native American; NHOPI, Native Hawaiian or Pacific Islander.

**Figure 4.**
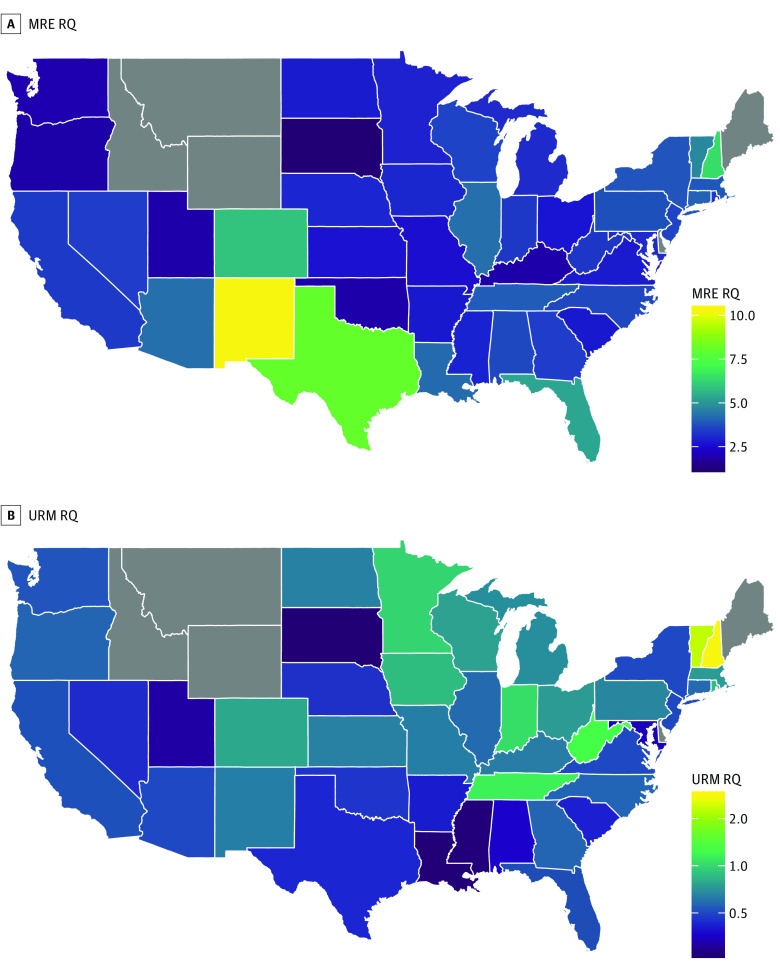
Choropleth Maps of Representation Quotient (RQ) for Underrepresented in Medicine (URM) Enrollees by State, 2017 to 2018 An RQ greater than 1 indicates that a subgroup is overrepresented among medical school enrollees relative to the US population, and an RQ less than 1 indicates that a subgroup is underrepresented. MRE indicates multiple race/ethnicity.

Total enrollment in the 2017 to 2018 school year was 89 789 in US allopathic medical schools. Figure 3 shows choropleth maps for RQ from the race/ethnicity-specific analyses by states, and Figure 4 shows the map for the composite URM category in a sensitivity analysis. States shown in gray either did not contain medical schools (Delaware, Idaho, Maine, Montana, and Wyoming) or had no enrollees identifying with the corresponding racial/ethnic group. The mean (SD) state-level RQ for individuals who self-identified as AIAN was 0.47 (0.49); black, 0.58 (0.58); Hispanic, 0.39 (0.32); NHOPI, 1.09 (1.75); Asian, 4.18 (1.96); white, 0.95 (0.22); and URM, 0.70 (0.47).

## Discussion

Using national AAMC data coupled with national and state-level US Census data, our study builds on prior work by assessing whether the medical student population (and thereby the future physician workforce) represents the US population it serves. Prior studies report modest improvements in diversity without accounting for such demographic population shifts. Boatright et al^6^ reported modest but statistically significant improvements in medical school matriculants for black and Hispanic students after the institution of the LCME diversity accreditation requirements. While this may be true with respect to the net number of black and Hispanic matriculants, it is not with respect to representation. We find no statistically significant trend toward increased representation for black and Hispanic male individuals and a modest trend toward increased representation for Hispanic female applicants. In fact, our results indicate that Hispanic individuals are underrepresented among medical school applicants and matriculants by nearly 70% relative to the age-adjusted US population; black male applicants and matriculants, nearly 60%; black female applicants, nearly 30%; and black female matriculants, nearly 40%. Similarly, AIAN individuals are underrepresented by more than 60% among applicants and matriculants. It is likely that the modest improvements in the proportion of minority matriculants reported by Boatright et al^6^ is due to the increase in the number of black and Hispanic individuals of the appropriate age to apply to or matriculate into medical school (eFigure 1 and eFigure 2 in the Supplement). Our findings suggest that black, Hispanic, and AIAN racial/ethnic groups remain underrepresented since the LCME policy enactment.

Guevara et al^8^ also reported modest improvements at the faculty level for minority groups without adjusting for changes in diversity in the US population. In contrast, our prior work revealed that the gap between the minority population in the academic physician workforce widened over time for nearly all specialties and faculty rankings.^11^ Our study examines physicians earlier in the pipeline and reveals a similar lack of improvements in racial/ethnic underrepresentation among both medical school applicants and matriculants.

We acknowledge that increasing diversity is better than decreasing diversity, irrespective of representation. However, if we are to achieve representation in accordance with LCME guidelines, these findings highlight an urgency to better measure and identify effective strategies to improve recruitment and retention of URM students. Given the duration of physician training, any changes implemented will take more than a decade to affect the physician workforce.

Underrepresentation of minority groups among physicians is a challenging problem with many contributing factors. This study shows that URM individuals are underrepresented among the applicant pool as well as the matriculant pool. It is well documented that interrelated societal elements such as lack of investment in public education,^12^ disparities in educational resource allocation,^13,14^ and de facto school segregation^15^ may limit the educational opportunities of minority populations, thus potentially detracting from the pipeline of these populations to medical school. However, little is known about the drivers and barriers for URM college graduates to apply to medical school, and it is unclear to what degree medical schools and their governing bodies contribute to or ameliorate barriers to this process. Future mixed-methods studies should explore the motivations for URM applicants to best identify targets for interventions.

Our cross-sectional analysis reveals stark differences in representation across racial/ethnic and geographic categories. As our map illustrates, populous states with significant diversity (eg, California, Texas, and Florida) are failing to train medical student cohorts whose composition is commensurate with their age-adjusted populations. In contrast, by virtue of a relatively smaller denominator, states with less diversity who can draw from a more diverse national applicant pool tend to have higher URM RQs. These findings reveal that there are large, untapped pools of potential URM applicants and matriculants in diverse states. However, it also suggests that strategies sensitive to local needs may be of particular benefit in improving representation. Such state-based strategies are especially important when considering that almost 40% of physicians practice in the state where they went to medical school and almost 50% practice in the state of their most recent graduate medical education.^16^

### Limitations

This study has some limitations. First, the change in the data collection method for the AAMC may have biased our race/ethnicity-specific results toward observing underrepresentation in URM groups, especially Hispanic students, owing to the exclusion of individuals with multiple racial and ethnic identities. However, our sensitivity analyses including the multiple racial/ethnic group category in a composite URM category did not significantly alter our primary findings, except for a significant trend toward decreased representation for URM female applicants from 2002 to 2012. It is important to note that this group includes individuals who are not URM, including individuals who self-identify as both Asian and white, thereby possibly overestimating the true number of URM individuals. Our analysis was also limited by the demographic categories of our data set. For example, our analysis shows consistent overrepresentation of Asian applicants and matriculants throughout the study period. However, the Asian racial/ethnic group has significant heterogeneity,^17,18,19^ with many within this group (eg, Laotian, Bangladeshi) who qualify as underrepresented. Our data sets also did not allow for distinguishing international matriculants as a subset of each race/ethnicity as well as the significant numbers of minority physicians who are trained in LCME-accredited medical schools in Puerto Rico. Additionally, we recognize that health care is delivered collaboratively with significant contributions from professionals without an MD degree. Assessing trends in diversity in the osteopathic medicine, nurse practitioner, and allied health professions pipelines will also be important in our efforts to achieve representation commensurate with the general population. In addition, our data set only includes sexual identities and does not account for broad gender diversity or account for other minority groups that should be considered when shaping an inclusive and representative physician workforce.^20^

## Conclusions

Nationally, from 2002 onward, we found a persistently deficient representation of black, Hispanic, and AIAN medical students. We also found that most states do not train physicians who are demographically representative of the surrounding population. Future evaluation can adapt the RQ to the institutional, regional, or state level to track diversity efforts with better resolution than previously reported national studies. While we constrained our cross-sectional analyses by age to be consistent with our longitudinal analyses, future inquiry could assess how state-level physician workforces reflect their general patient population, irrespective of age.

Achieving representation will alter the current makeup of our physician workforce and therefore has inherent political implications. We do not advocate for implementation of specific quotas or target proportions by group, as such policies would not effectively accommodate the dynamic nature of the US population. However, given the mounting evidence that diversifying the workforce to reflect the population served is key to providing high-quality, high-value, culturally effective care,^3,5,21^ we have an evidence-based imperative to find more effective policies to promote representation.

## Notes

Supplement. **eFigure 1.** Counts by Race/Ethnicity and Sex for Medical School Applicants, 2002-2017 **eFigure 2.** Proportions by Race/Ethnicity and Sex for Medical School Applicants, 2002-2017 **eFigure 3.** Counts by Race/Ethnicity and Sex for Medical School Matriculants, 2002-2017 **eFigure 4.** Proportions by Race/Ethnicity and Sex for Medical School Matriculants, 2002-2017
