# Supplementary material for: Trends in Racial/Ethnic Representation Among US Medical Students
Source: JAMA Netw Open. 2019 Sep 4;2(9):e1910490. doi: 10.1001/jamanetworkopen.2019.10490 (PMC6727686; doi:10.1001/jamanetworkopen.2019.10490)
Supplement: Supplement. — eFigure 1. Counts by Race/Ethnicity and Sex for Medical School Applicants, 2002-2017 eFigure 2. Proportions by Race/Ethnicity and Sex for Medical School Applicants, 2002-2017 eFigure 3. Counts by Race/Ethnicity and Sex for Medical School Matriculants, 2002-2017 eFigure 4. Proportions by Race/Ethnicity and Sex for Medical School Matriculants, 2002-2017 [file jamanetwopen-e1910490-s001.pdf]

Supplementary Online Content

Lett E, Murdock HM, Orji WU, Aysola J, Sebro R. Trends in racial/ethnic representation among US medical students. *JAMA Netw Open*. 2019;2(9):e1910490. doi:10.1001/jamanetworkopen.2019.10490

eFigure 1. Counts by Race/Ethnicity and Sex for Medical School Applicants, 2002-2017
eFigure 2. Proportions by Race/Ethnicity and Sex for Medical School Applicants, 2002-2017
eFigure 3. Counts by Race/Ethnicity and Sex for Medical School Matriculants, 2002-2017
eFigure 4. Proportions by Race/Ethnicity and Sex for Medical School Matriculants, 2002-2017

This supplementary material has been provided by the authors to give readers additional information about their work.

© 2019 Lett E et al. *JAMA Network Open.*

eFigure 1: Counts by Race/Ethnicity and Sex for Medical School Applicants, 2002-2017

Applicant Counts, 2002–2017

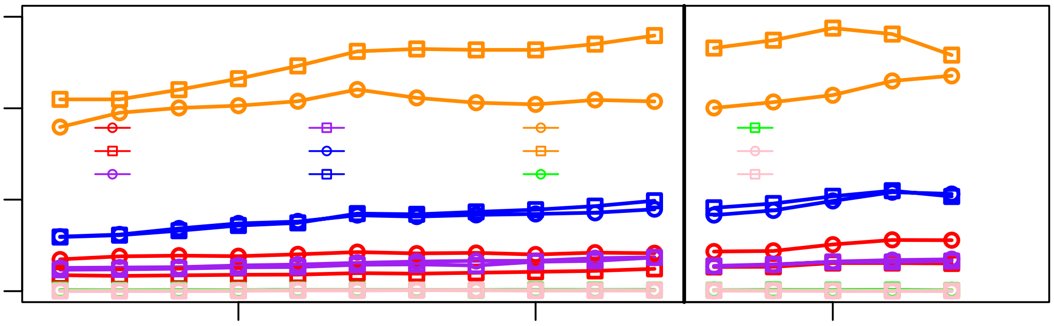

Applicant Counts, 2002–2017

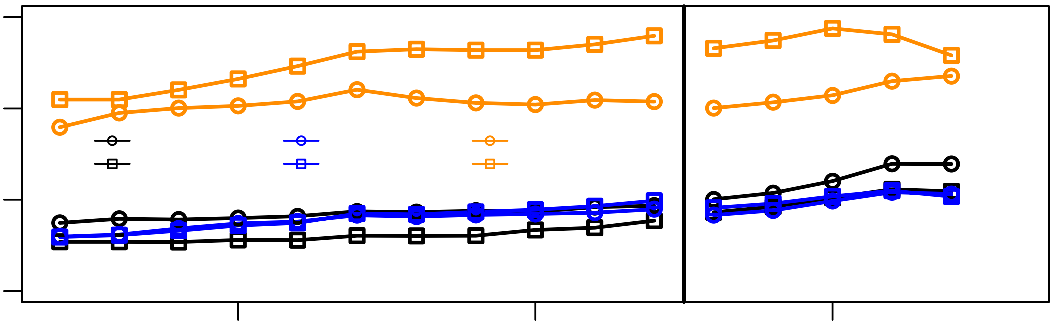

eFigure 1 legend:
AIAN - American Indian or Alaska Native
NHOPI - Native Hawaiian or Other Pacific Islander
URM – Under-represented in medicine (Black, Hispanic, AIAN, NHOPI, Two or more races)
Red line with red squares – Black males
Red line with red circles – Black females
Purple line with purple squares – Hispanic males
Purple line with purple circles – Hispanic females
Blue line with blue squares – Asian males

© 2019 Lett E et al. *JAMA Network Open.*

Blue line with blue circles – Asian females
Orange line with orange squares – White males
Orange line with orange circles – White females
Green line with green squares – AIAN males
Green line with green circles – AIAN females
Pink line with pink squares – NHOPI males
Pink line with pink circles – NHOPI females
Black line with black squares – URM males
Black line with black circles – URM females

© 2019 Lett E et al. *JAMA Network Open.*

eFigure 2: Proportions by Race/Ethnicity and Sex for Medical School Applicants, 2002-2017

Applicant Proportions, 2002–2017

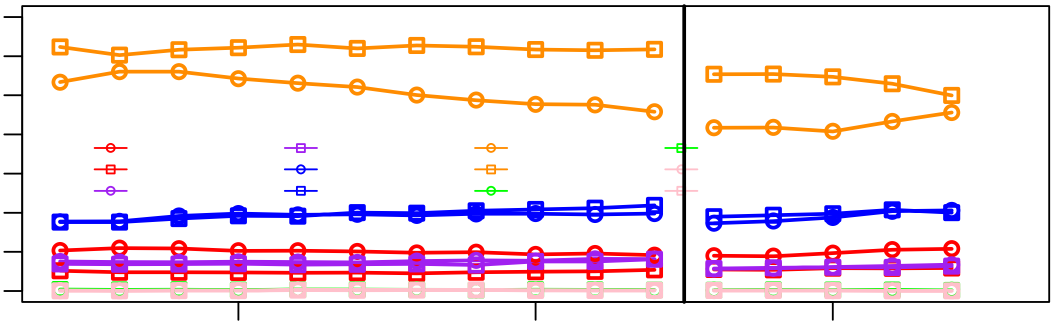

Applicant Proportions, 2002–2017

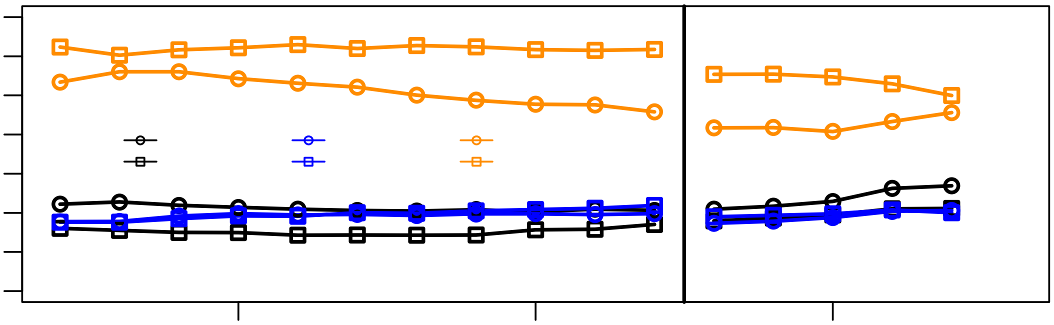

eFigure 2 legend:
AIAN - American Indian or Alaska Native
NHOPI - Native Hawaiian or Other Pacific Islander
URM – Under-represented in medicine (Black, Hispanic, AIAN, NHOPI, Two or more races)
Red line with red squares – Black males
Red line with red circles – Black females
Purple line with purple squares – Hispanic males
Purple line with purple circles – Hispanic females

© 2019 Lett E et al. *JAMA Network Open.*

Blue line with blue squares – Asian males
Blue line with blue circles – Asian females
Orange line with orange squares – White males
Orange line with orange circles – White females
Green line with green squares – AIAN males
Green line with green circles – AIAN females
Pink line with pink squares – NHOPI males
Pink line with pink circles – NHOPI females
Black line with black squares – URM males
Black line with black circles – URM females

© 2019 Lett E et al. *JAMA Network Open.*

eFigure 3: Counts by Race/Ethnicity and Sex for Medical School Matriculants, 2002-2017

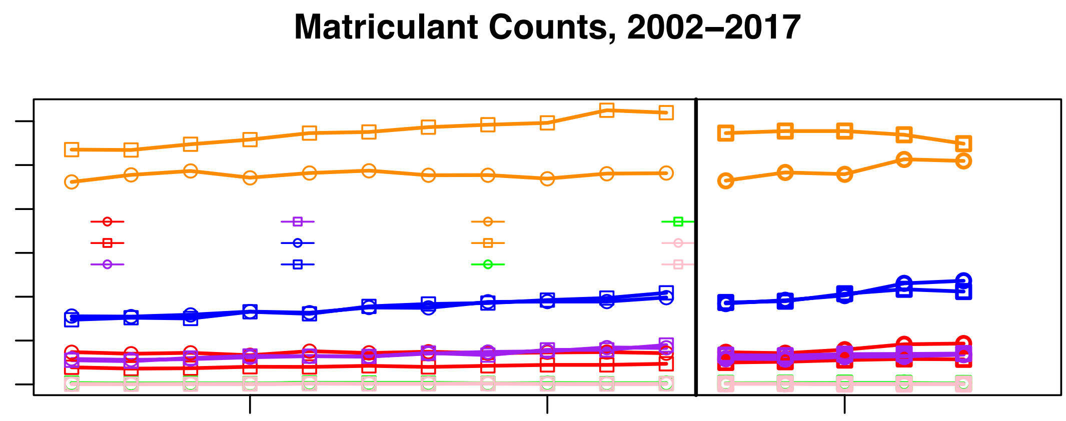

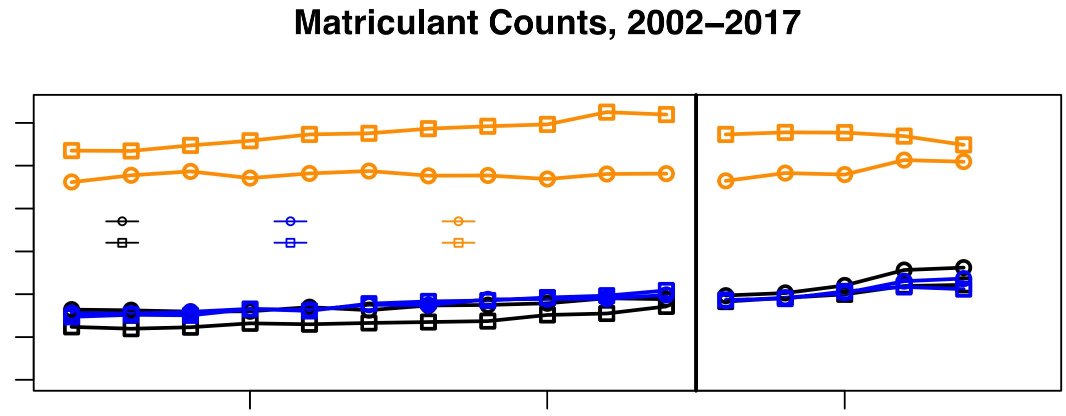

eFigure 3 legend:
AIAN - American Indian or Alaska Native
NHOPI - Native Hawaiian or Other Pacific Islander
URM – Under-represented in medicine (Black, Hispanic, AIAN, NHOPI, Two or more races)
Red line with red squares – Black males
Red line with red circles – Black females
Purple line with purple squares – Hispanic males
Purple line with purple circles – Hispanic females
Blue line with blue squares – Asian males
Blue line with blue circles – Asian females
Orange line with orange squares – White males

© 2019 Lett E et al. *JAMA Network Open.*

Orange line with orange circles – White females
Green line with green squares – AIAN males
Green line with green circles – AIAN females
Pink line with pink squares – NHOPI males
Pink line with pink circles – NHOPI females
Black line with black squares – URM males
Black line with black circles – URM females

© 2019 Lett E et al. *JAMA Network Open.*

eFigure 4: Proportions by Race/Ethnicity and Sex for Medical School Matriculants, 2002-2017

Matriculant Proportions, 2002–2017

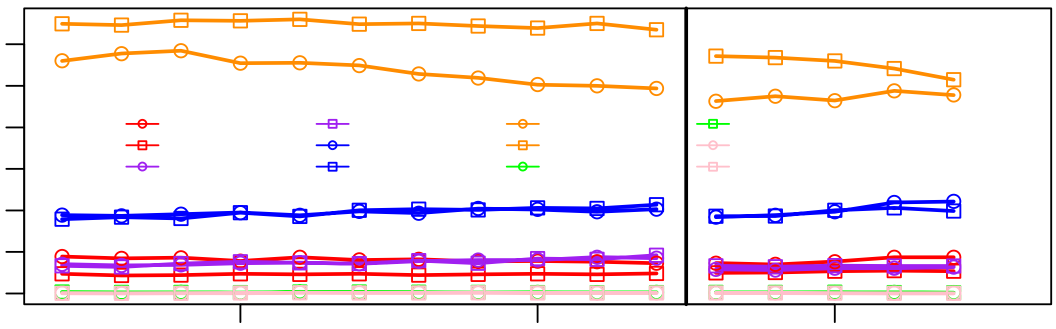

Matriculant Proportions, 2002–2017

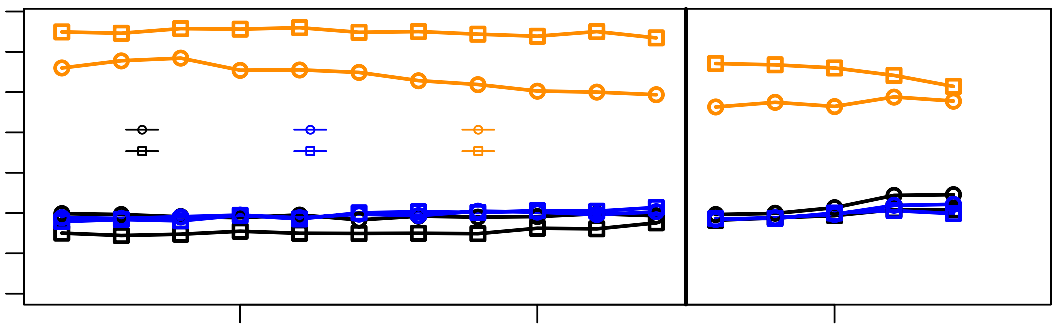

eFigure 4 legend:
AIAN - American Indian or Alaska Native
NHOPI - Native Hawaiian or Other Pacific Islander
URM – Under-represented in medicine (Black, Hispanic, AIAN, NHOPI, Two or more races)
Red line with red squares – Black males
Red line with red circles – Black females
Purple line with purple squares – Hispanic males
Purple line with purple circles – Hispanic females
Blue line with blue squares – Asian males

© 2019 Lett E et al. *JAMA Network Open.*

Blue line with blue circles – Asian females
Orange line with orange squares – White males
Orange line with orange circles – White females
Green line with green squares – AIAN males
Green line with green circles – AIAN females
Pink line with pink squares – NHOPI males
Pink line with pink circles – NHOPI females
Black line with black squares – URM males
Black line with black circles – URM females

© 2019 Lett E et al. *JAMA Network Open.*
